# Neglected intrapelvic dislocation of femoral head

**DOI:** 10.4103/0019-5413.61727

**Published:** 2010

**Authors:** Vaidynathan Singaravadivelu, Moongilpatti Sengodan Mugundhan, Karunanandaganapathy Sankaralingam

**Affiliations:** Department of Orthopedics, Kilpauk Medical College, Chennai, India

**Keywords:** Dislocation of hip, intrapelvic dislocation of hip, cemented total hip arthroplasty

## Abstract

A 40-year-old man sustained trauma while walking and presented three months after trauma. The attitude of left hip was flexion, neutral rotation, and neutral abduction/adduction with an arc of flexion available from 30° to 90°. No movement was possible in other planes. On roentgenogram and 3D computed tomography, the femoral head was lying inferior and medial to the acetabulum, and it was in the same coronal plane of the acetabulum, neither anterior nor posterior, within the pelvic cavity. An indentation over the superolateral half of femoral head was observed. The acetabulum was found intact except for a small defect in the inferior aspect of its medial wall. Cemented total hip replacement was done without any need to reconstruct the acetabulum, and at the two years follow-up, the patient was having a pain-free, stable hip and was able to walk unaided. We hereby report a late presentation of intrapelvic dislocation of hip in view of rarity.

## INTRODUCTION

Hip dislocation has been classified as anterior and posterior according to the position of femoral head, with reference to the plane of acetabulum. Central dislocation, though considered a misnomer by some authors, has been described as a medial displacement of femoral head secondary to displaced acetabular fracture.^1^ Chung describes medial or intrapelvic dislocation as tearing of joint capsule medially with dislocated femoral head lying medial to the pubic bone. This may be accomplished by acetabular fracture and rupture of bladder.^2^

We report a case of intrapelvic dislocation, wherein the femoral head was lying inferior to acetabulum, with no associated fracture in the femur. The acetabulum was also found intact except for a small defect in the inferior aspect of its medial wall.

## CASE REPORT

A 40-year-old man had a fall while walking three months ago. He was not able to stand after the trauma. He was treated by a traditional bone setter with a topical medication without any manipulation or splinting. He started walking after 1.5 months, using a stick.

Three months later, he presented with a painful left hip and an inability to bear weight on the left lower limb. On examination, both his anterior superior iliac spines were at the same level. The left lower limb was in flexion, neutral rotation, and neutral in abduction/adduction plane. The only available movement was flexion from 30° to 90°.

**Figure 1:**
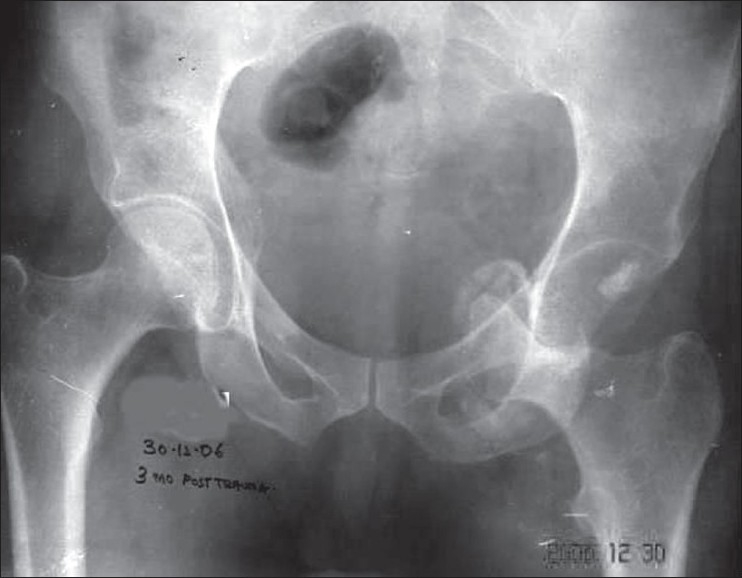
Preoperative anteroposterior X-ray of pelvis with both hips showing intra pelvic dislocation

**Figure 2:**
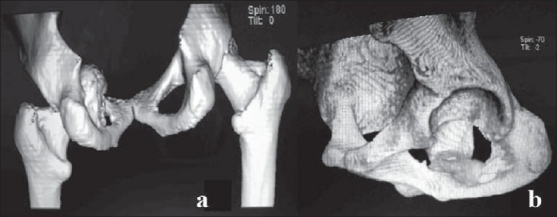
(a) 3D reconstructed CT, viewed from behind depicts the intrapelvic dislocation of left hip. (b) Subtracted 3D reconstructed CT image showing the integrity of the left acetabulum with a defect in the inferior aspect of medial wall

**Figure 3:**
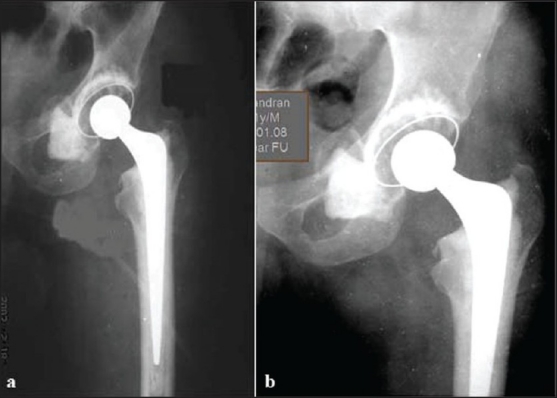
Immediate postoperative (a) and one year followup (b) X-ray of left hip showing cemented total hip arthroplasty

Roentgenogram [Figure 1] and 3D computed tomography [Figure 2] confirmed the plane of dislocation. The femoral head was lying inferior to and in the same plane of acetabulum, within the pelvic cavity [Figure 2a]. There was an indentation over the superolateral half of femoral head, with no fracture in the femur. Correspondingly, there was a defect in the inferior aspect of medial wall of acetabulum which was confirmed on 3D subtraction image [Figure 2b]. However, there was no corresponding fracture fragment found in the images.

The patient was operated and total hip replacement was done through a posterior approach. Fibrous tissue covering the acetabulum was cleared. The head was lying inferior to the acetabulum and was found within the pelvis in a pseudocapsule. The head was delivered out by flexion, abduction, internal rotation, and lateral pull. There was a smooth indentation over the superolateral half of femoral head. The transverse acetabular ligament was found torn. The acetabulum was also found intact except for a small defect in the inferior aspect of its medial wall. Cemented total hp replacement was done using Exeter total hip system (Howmedica Inc.).

Postoperative roentgenogram revealed well-placed components with excess cement lying in the pseudocapsule within the pelvis [Figure 3]. The patient started walking with support on the second postoperative day. He was able to do active straight leg raising and abduction against gravity by the second week. At the 2 years follow-up, the patient was walking unaided with a stable and pain-free hip.

## DISCUSSION

Polesky *et al.* reported a case of iatrogenic intrapelvic dislocation of femoral head with a subcapital fracture in the neck of the femur sustained while reducing traumatic anterior dislocation of the hip.^3^

Shohayeb *et al*. reported a case of intrapelvic dislocation of the head of the femur through obturator foramen by means of fracture of the inferior pubic ramus, associated with the ipsilateral fracture of the femur and greater trochanter.^4^

A case of central acetabular fracture with ipsilateral femoral neck fracture and intrapelvic dislocation of the femoral head without major pelvic column disruption was reported by Meinhard *et al.* On reviewing the literature, all patients who had intrapelvic displacement of the femoral head with transcervical fracture were associated with other pelvic fractures particularly fractures of anterior column, posterior column, and dome fracture.^5^

All the reported cases of intrapelvic dislocations have been associated with the fracture of the ipsilateral femur such as the femoral neck, trochanter, shaft, or acetabulum. In our case, except for a smooth indentation over the superolateral half of the femoral head and a corresponding defect in the inferior aspect of medial wall of the acetabulum, no fracture was detected in the ipsilateral femur, acetabulum, and pelvic column.

The mechanism of injury in all the cases reported so far was major road traffic accidents, whereas in our patient, it was only a domestic fall. Because the patient presented to us three months after trauma, no immediate posttraumatic X-ray or 3D CT were available, and therefore, the exact mechanism of injury could not be ascertained. Possibly the initial injury would have been an obturator type of anterior dislocation with an impaction fracture in the femoral head. On continued neglect, the femoral head might have migrated to become intrapelvic in location.

Jamshid *et al*. studied the osteochondral impaction of the femoral head in dislocations on CT scan (n=35) and reported that the impaction was found in 67% of posterior and in 100% of anterior dislocations. In the case of anterior dislocation, the impaction of the femoral head occurred in posterosuperior and lateral portion of the femoral head. The lesion is analogous to Hillsach's lesion in shoulder.^6^

Robert *et al*. observed that the impaction in obturator dislocation was due to the impingement against the anteroinferior margin of acetabular rim.^7^ In our case, the impaction involved the entire superolateral half of the femoral head, indicating that the inferior margin of acetabulum was the cause for impingement.

The incidence of neglected dislocation is still prevalent in developing countries, majority of which are posterior hip dislocations and very rarely anterior dislocation.^8^ Neglected intrapelvic dislocation has not been reported in the English literature so far to the best of our knowledge.
